# Plant–Soil Feedbacks May Underlie Competitive Dynamics Between Co‐Occurring Invasive Annual Grasses *Bromus tectorum* and *Ventenata dubia*

**DOI:** 10.1002/ece3.74516

**Published:** 2026-10-18

**Authors:** Lilly Sencenbaugh, Jane M. Mangold, Bruce D. Maxwell, Danielle E. M. Ulrich, Lisa J. Rew

**Affiliations:** ^1^ Land Resources and Environmental Sciences Montana State University Bozeman Montana USA; ^2^ Montana State University, Ecology Bozeman Montana USA

**Keywords:** *Bromus tectorum* (cheatgrass), competition, invasion ecology, invasive annual grass, plant–soil feedback, *Ventenata dubia* (ventenata North Africa grass)

## Abstract

Plant communities are shaped by interactions among plants and soil legacies. Plant–soil feedbacks (PSF) may alter invasion dynamics and change plant community species composition. There have been field observations of the invasive annual grass, *Ventenata dubia* , replacing another invasive annual grass, *Bromus tectorum* , though previous work has not found evidence of competition that would support these shifts. The goal of this study was to determine if PSF via soil legacies and competition are present between co‐occurring invasive annual grasses *B. tectorum* and *V. dubia* , and if so, quantify their effects on interactions with these species. We measured aboveground per capita biomass of these species grown in biculture with three field soils (neutral, *B. tectorum* , *V. dubia* ) over two experimental Phases in a greenhouse. We evaluated biomass in Phase 1 with field‐conditioned soil, and Phase 2 in soil conditioned from Phase 1. We used generalized linear mixed models to determine the effect of soil origin and plant density on biomass, and non‐linear analysis to quantify competition regression coefficients. The linear analysis showed intraspecific competition negatively impacted both species in both Phases, but by Phase 2 interspecific competition was weakened in conspecific soil, suggesting positive PSF. Intraspecific competition coefficients were always greater than interspecific coefficients from the non‐linear analysis across soil origins. These results are suggestive of a positive PSF which may alter the strength of competition between these species in a biculture. Understanding how PSF influences interactions between these species provides insight into how their invasion dynamics may change over time.

## Graphical abstract

Plant communities are continuously shaped by interactions between plants and the soil microbial community, which may be influential in plant invasions. We conducted a plant‐soil feedback and competition study on two problematic invasive annual grasses that are of major concern in North American rangelands. We found evidence suggestive of positive feedbacks with these species that may alter their competitive dynamics and be conducive toward formation of monotypic stands in invaded range.

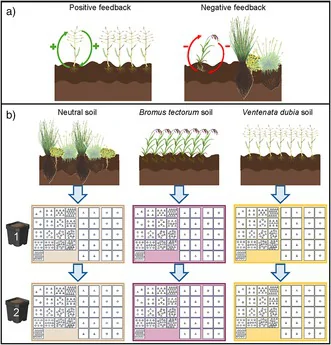

## 1 Introduction

Interactions shape the composition of plant communities (Tilman 1982; Levine et al. 2017; Saavedra et al. 2017), and interactions among invasive species may alter the dynamics of their invasion (Kuebbing et al. 2013; Kuebbing and Nuñez 2015; Pearson, Ortega, Runyon, and Butler 2016; Oduor et al. 2024). Competitive interactions between invaders may be conducive to secondary invasion, as removal of a competitor (e.g., through management) could release the secondary invader from competitive pressure, thus allowing for a subsequent increase in its population density or range (Pearson, Ortega, Runyon, and Butler 2016; Oduor et al. 2024). Simultaneously, soil properties and microbial communities influence plant fitness and productivity (Bever et al. 1997; Wardle et al. 2004), which may also impact the strength and direction of invasion as successive invaders may benefit from changes made to soil properties and microbial communities (Reinhart et al. 2003; Suding et al. 2013; Zhang et al. 2020). These plant–soil interactions may change plant communities by altering the fitness of a plant species or by affecting niche differences among plant species (Bever 1994; van der Putten et al. 2013; Lankau and Keymer 2018; Ke and Wan 2020).

Plant–soil feedbacks (PSF) are the interactions between plants and the soil, where plants impact soil abiotic or biotic properties, which therefore impact plant fitness and productivity (Bever et al. 1997; van der Putten et al. 2013). PSF may impact co‐occurring invasive species, and differences in soil legacy effects may alter the fitness of one invader more than another, affecting plant productivity and competitive interactions (Chisholm and Muller‐Landau 2011; Crawford and Knight 2017; Zhang et al. 2020; Chen and van Kleunen 2022). Positive soil legacy effects have been documented between different invasive species, in which invasive plants may increase in competitive ability (Zhang et al. 2020) or productivity (Chen and van Kleunen 2022) in response to the soil being previously occupied by a different invader, as opposed to a native community. When PSF is associated with an apparent increase in plant fitness, there is a “positive feedback” that can correspond with a decrease in intraspecific competition, resulting in an increase in the number of individuals of the same species (Lee et al. 2012; van der Putten et al. 2013). In the opposite scenario, a “negative feedback” is when the plant species has an apparent decrease in fitness and demonstrates increased intraspecific competition, resulting in a decrease in the number of individuals of the same species (Mangan et al. 2010; Bever et al. 2015; Crawford et al. 2019; Figure 1a). The greater intraspecific competition relative to interspecific competition associated with negative PSF is thought to be more conducive to multiple species coexistence than positive PSF (Chesson 2000; Letten et al. 2017; Barabás et al. 2018; Lekberg et al. 2018; Zhang et al. 2020). Coexistence may occur due to niche differences that reduce competitive exclusion, which is in part determined by fitness differences like reproductive output (Chesson 2000; Barabás et al. 2018; Ke and Wan 2020), thus, negative PSF may promote more even species distributions (increased alpha and beta diversity) (Mangan et al. 2010; Chisholm and Muller‐Landau 2011; Lekberg et al. 2018). Contrastingly, positive PSFs may be related to communities with dense monocultures or clumping patterns of individual species (Bennett et al. 2017): patterns often associated with invasive species (Crawford and Knight 2017).

**FIGURE 1:**
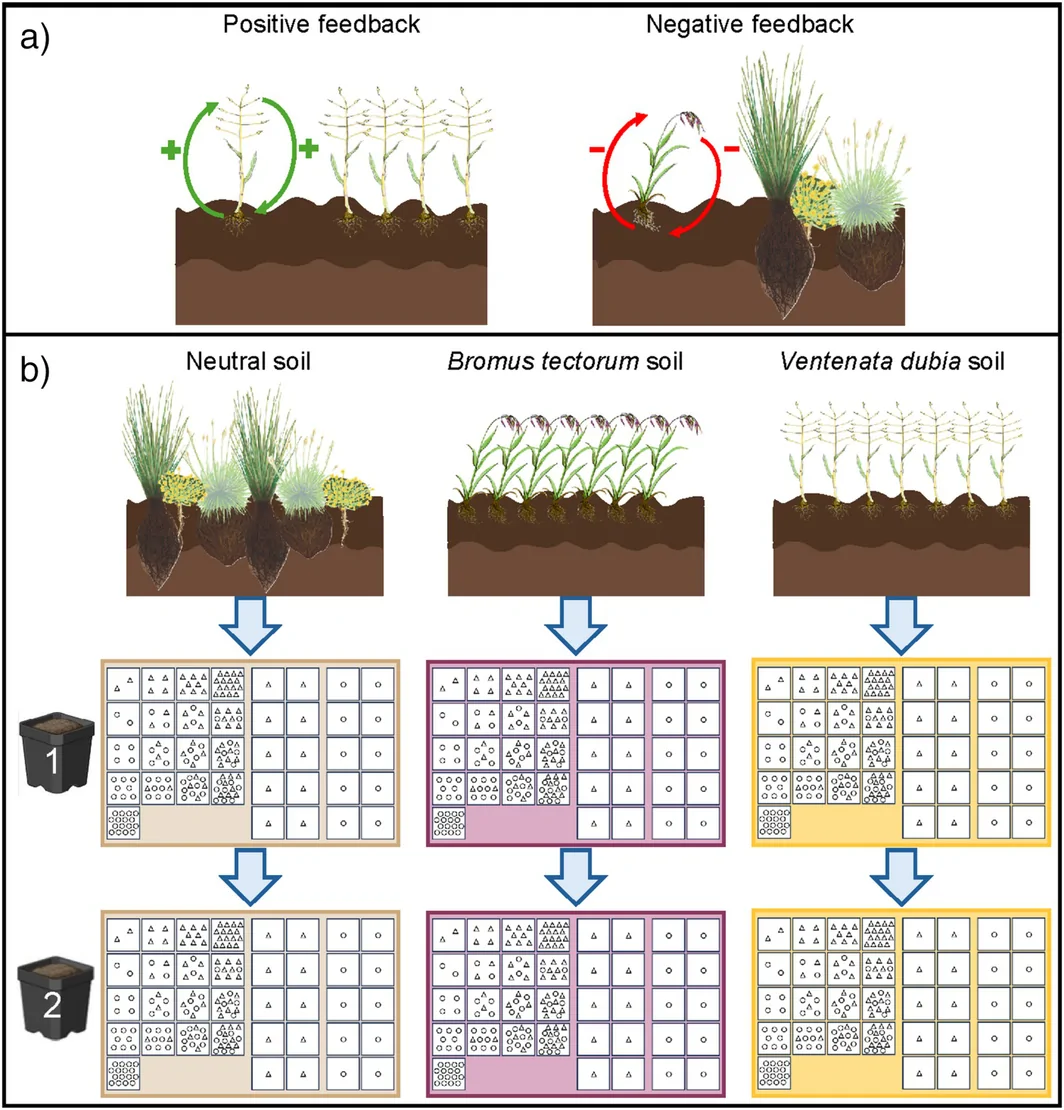
Diagram of plant–soil feedback (PSF) and experimental design. In all panels the brown box represents the soil, in which microorganisms, soil nutrient content, or other factors may influence PSF. Panel a illustrates a PSF, where greater density of the target plant may be sustained due to positive feedback (green arrows) and a negative PSF (red arrows), where the target plant is density dependent due to soil feedback limiting fitness. The plants demonstrating feedbacks are non‐specific invasive annual grasses. The patterns shown in the panels are not demonstrations of results from our studies or others, rather illustrations of potential interactions. Panel b is a diagram of our experimental design. Field soils were delineated as: “Neutral” areas historically confirmed to not be invaded by *B. tectorum* nor *V. dubia* ; “ *B. tectorum* ” areas invaded by *B. tectorum* but not *V. dubia* ; and “ *V. dubia* ” areas that were invaded by *V. dubia* but not *B. tectorum* . Pots were filled with pasteurized potting soil and topped with a 1 cm layer of field soil. Seeds were sown in an addition series matrix (represented by white boxes) with 10 singles grown of each species (circle representing *B. tectorum* and triangle representing *V. dubia* , not to scale). The addition series was repeated four times within each soil treatment to a total of 324 pots (17 competition arrangements * 3 soils * 4 reps +10 individuals * 3 soils * 4 reps). After conclusion of phase 1 (10 weeks), plants were harvested and the pots were left dry for two weeks. After two weeks the experiment was conducted again (phase 2) in which the same competition arrangement was planted into the same corresponding pot from phase 1.

When considering invasive species, it is important to quantify the strength and direction of interactions between co‐occurring invaders so that we can understand how plant community assembly may change over time, as interactions between invaders may amplify or weaken the effects of an invasion and alter responses to management (Kuebbing et al. 2013; Kuebbing and Nuñez 2015). Further, improving understanding of PSF on competing invaders will enhance predictions of invasion dynamics and competitive interactions (Crawford and Knight 2017; Zhang et al. 2020). It has been suggested that semi‐arid rangelands in the American West are being negatively impacted by the invasion of non‐native annual grasses, including *Bromus tectorum* L. (cheatgrass, downy brome) and *Ventenata dubia* (Leers) Coss (North Africa grass, ventenata) (DiTomaso 2000; Germino et al. 2015; Brummer et al. 2016; Pearson, Ortega, Eren, and Hierro 2016; Fryer 2017). These species can form dense monocultures (Reisner et al. 2013; Wallace et al. 2015; Jones et al. 2018), decrease native species diversity (Jones et al. 2020; De Stefano et al. 2024), limit quality of forage for livestock and wildlife (Brummer et al. 2023), and have varying effects on soil microbial communities, notably AM fungi (Busby et al. 2012; Lekberg et al. 2013; Tortorelli et al. 2022; Sullivan et al. 2023; Berner et al. 2025).

Both *B. tectorum* and *V. dubia* are cool season grasses that germinate in the fall (Mack 1981; Wallace and Prather 2016; Fryer 2017), and typically to a lesser extent in the spring, though *V. dubia* does have a significant spring cohort in Western rangelands (Wallace et al. 2015). Whether these species overlap in the field is dependent on abiotic factors like soil moisture and elevation (Jones et al. 2018, 2020). While there has been anecdotal evidence of *V. dubia* replacing *B. tectorum* populations (Barkworth et al. 1993; Northam and Callihan 1994; Prather and Burke 2011), such replacement is not likely to be caused by aboveground competition alone based on previous competition study results (Harvey et al. 2020; Sencenbaugh et al. 2025). However, management and small differences in the species growth habits may contribute to changes in the competitive balance. Secondary invasion occurs following the targeted removal (Pearson, Ortega, Runyon, and Butler 2016) of a dominant invader that often has different niche dimensions, for example in terms of phenology or life form (Lesica and Hanna 2009; Larson and Larson 2010; Pearson, Ortega, Runyon, and Butler 2016).

Given the similarities between *B. tectorum* and *V. dubia* we would expect analogous management responses (Pearson, Ortega, Runyon, and Butler 2016). A common management strategy for invasive annual grasses is fall applications of pre‐ or early post‐emergent herbicides including those with the active ingredients indaziflam or imazapic (Mangold et al. 2013; Monaco et al. 2017; Sebastian et al. 2017), both of which have demonstrated effects on *B. tectorum* and *V. dubia* (Sebastian et al. 2016; Mangold et al. 2024). While these species are likely to be managed concurrently using these methods, it is possible that fall applications of herbicides with shorter residual activity (i.e., imazapic) may be ineffective toward spring germinating cohorts of *V. dubia* , thus contributing to secondary invasion of this species. Given that both species occupy similar habitats (Bradley 2009; Taylor et al. 2014; Wallace et al. 2015; Jones et al. 2020) and are potentially increasing in range overlap due to climatic changes (Williamson et al. 2020; Adhikari et al. 2022; Nietupski et al. 2024), there may be more opportunities for future interactions.

Competitive interactions between these species have varied across studies, so it is unclear whether either species has an aboveground competitive advantage over the other (Harvey et al. 2020; Sencenbaugh et al. 2025), but the role of PSF in causing varying competitive outcomes has not been studied. However, there is evidence of soil legacy effects, often attributed to the earlier senescence and shallower rooting of these species relative to native perennial grasses (Germino et al. 2015; Fryer 2017). *Bromus tectorum* litter has a greater C:N and lignin:N ratio than many native perennial species, which contributes to the alteration in nitrogen availability following decomposition (Rickard 1985). In *B. tectorum* invaded rangelands, this may alter nitrogen cycling, as *B. tectorum* acquires and assimilates the subsurface nitrogen, which is ultimately deposited on the surface as litter; then decomposition leads to nitrogen deposition back into the subsurface to ultimately be acquired by *B. tectorum* (Evans et al. 2001; Sperry et al. 2006). Alteration of litter cover in semi‐arid rangeland systems can lead to a decrease in biological soil crusts which are important for nitrogen mineralization (Evans et al. 2001). Beyond altering the quantity and quality of litter, regions affected by *B. tectorum* invasion often have a shift in soil resource partitioning due to the transition from a diverse community of deep or tap rooting species to *B. tectorum* 's shallow fibrous roots (Wilcox et al. 2012; Germino et al. 2016). *Ventenata dubia* potentially alters the quantity and quality of litter due to high silica content in leaves resulting in slower litter decay than low silica organic matter (Brummer et al. 2023).

Changes to the soil due to these species have also been linked to AM fungi, where studies have shown that *B. tectorum* has a negative or neutral effect on AM fungi abundance in areas it has invaded (Busby et al. 2012; Lekberg et al. 2013; Cheeke et al. 2021), and the effect of *V. dubia* varies depending on study location (Sullivan et al. 2023). Changes to the AM fungi, soil nutrient cycling, and litter content are all related components that may ultimately cause a PSF (Vogelsang and Bever 2009; Lee et al. 2012; Freschet et al. 2013; van der Putten et al. 2016; Sullivan et al. 2023; Oduor et al. 2024). Understanding the role of PSF on the invasion, competition, soil legacy, and assembly of these species will improve predictions of future invasion in western semi‐arid rangelands.

The main goals of this research were to determine if invasive annual grasses *B. tectorum* and *V. dubia* demonstrate any responses to soils conditioned by previous generations of the same species, the opposite species, or neither, and if the soil changes the competitive outcomes between the species. This research focuses on the soil legacy aspects of PSFs and does not examine microbes specifically, though future work would ideally quantify these interactions. To address the soil legacy aspects of plant–soil feedbacks, we conducted an addition series study in three soils with different plant occupancy legacies (neutral, *B. tectorum* , and *V. dubia* ) over two 10‐week growth periods (Phases 1 and 2) under controlled greenhouse conditions. We evaluated the aboveground biomass per individual plant (per capita, hereafter) to determine the impacts of soil origin and density of conspecifics (same species) or allospecifics (different species), and calculated competition coefficients for both species.

We hypothesized that both species would demonstrate competitive outcomes suggestive of a positive PSF due to their tendencies to form dense monocultures in the field (Reisner et al. 2013; Wallace et al. 2015; Jones et al. 2018, 2020; De Stefano et al. 2024). Due to anecdotal observations of *V. dubia* dominating sites formerly invaded by *B. tectorum* (Barkworth et al. 1993; Northam and Callihan 1994; Prather and Burke 2011), we hypothesized that *B. tectorum* conditioned soil would have a positive effect on *V. dubia* , but that *V. dubia* conditioned soil would have a negative effect on *B. tectorum* . A negative effect of *V. dubia* soil on *B. tectorum* individuals would be evident through *B. tectorum* biomass being the same or smaller in the *V. dubia* soil relative to the *B. tectorum* conditioned soil, which could explain dominance by *V. dubia* in fields once dominated by *B. tectorum* . With respect to competition, we hypothesized that interspecific competition coefficients would be greater than intraspecific, based on previous competition studies which have found this pattern (Sencenbaugh et al. 2025), and that neither species would have an obvious advantage over the other based on aboveground competition alone (Harvey et al. 2020; Sencenbaugh et al. 2025) or PSF. Greater intraspecific competition (relative to interspecific) has been suggested to be a mechanism conducive to multispecies competition (Chesson 2000; Letten et al. 2017; Barabás et al. 2018), thus greater interspecific competition may further explain the dense monocultures observed in these species in the field (Reisner et al. 2013; Wallace et al. 2015; Jones et al. 2018, 2020; De Stefano et al. 2024). Finally, we also posited that competition outcomes between species may be suggestive of a PSF, as positive direct PSF may be demonstrated through biomass being less impacted by competition in conspecific soil. Competition coefficients would reflect these patterns, where the coefficients would be smaller in conspecific soil relative to the neutral and allospecific soils. All together these patterns may implicate PSF as a possible mechanism underlying the competition‐based outcomes of these invasive annual grasses.

## 2 Methods

### 2.1 Experimental Design

Soil (Meagher loam) samples were collected to a depth of 10 cm in a field near Springhill Community in Gallatin County, MT (45° 52′ 44″, W 111° 03′ 57″) on November 11, 2021 (Web Soil Survey 2025). We collected soil from three regions within 100 m of each other: “neutral” areas historically confirmed to not be invaded by *B. tectorum* nor *V. dubia* ; “ *B. tectorum* ” areas invaded by *B. tectorum* but not *V. dubia* ; and “ *V. dubia* ” areas that were invaded by *V. dubia* but not *B. tectorum* . We did not include a sterilized soil control; therefore, we cannot separate microbial from abiotic drivers of soil effects; rather, we interpret differences among soil treatments as whole effects. In the greenhouse, pots (15 cm diameter and 11 cm deep, 1767 cm^3^) were filled with pasteurized (70°C for 60 min) potting soil that was equal parts loam soil, washed concrete sand, and Canadian Sphagnum peat moss. Then a layer of field soil, either from the neutral, *B. tectorum* invaded, or *V. dubia* invaded areas, was spread over the potting soil to a depth of 1 cm. Seeds were planted in the first 0.5 cm of field soil and grown for two, 10 week periods in the Montana State University Plant Growth Center (N 45°40′ 05.3″, W 111°03′ 12.0″) greenhouse (light/dark, 18°C–29°C/10°C–23°C, MVR1000/C/U Multi‐Vapor bulbs GE Lighting) and watered every other day to maintain soil moisture. Pots were over seeded by two individuals (per species if in biculture) and thinned. Extra seedlings were grown in greenhouse trays prepared with each soil type using the same methods as above, with wide spacing between individuals. If a pot did not have enough seedling emergence by week two, a seedling from the trays was planted. Two weeks following the harvest of Phase 1, new seeds were sown for Phase 2 (Figure 1b).

*Bromus tectorum* seeds were collected near Norris, MT, from Red Bluff Research Ranch (N 45° 33′ 1.85121″, W 111° 39′ 30.62804″). *Ventenata dubia* seeds were composited from collections near Bozeman, MT (N 45° 45′ 32.3″, W 111° 08′ 39.3″); Lodge Grass, MT (N 45° 16′ 17.4″, W 107° 35′ 16.9″); and Missoula, MT (N 46° 53′ 55.6″, W 113° 56′ 58.3″). For both Phases of the experiment, individual plants were harvested for aboveground biomass after the 10‐week growth period. Biomass samples were placed in coin envelopes and dried for 48 h at 37°C and weighed to the nearest 0.0001 g.

Rather than implementing a full factorial PSF design, this study evaluated how conditioning from previous soil occupancy influences the competitive dynamics of these species through an addition series matrix design to evaluate competitive dynamics in the three soil types (see Figure 1b). Both species had five density levels (1, 2, 4, 8, and 16), where the middle densities (2, 4, and 8) were grown in monoculture and biculture, and the minimum and maximum densities (1 and 16) were in monoculture exclusively, though few pots maintained the desired 16 plants to the end of 10 weeks for either Phase. These densities were selected following Sencenbaugh et al. (2025) and review of other competition studies that included one or more of these species (Larson et al. 2018; Harvey et al. 2020), so that middle densities were high enough to provoke a density‐dependent response. Each competition arrangement was repeated 4 times within each soil treatment for a total of 204 pots (17 competition arrangements * 3 soils * 4 reps). Simultaneously, 10 individual plants per species were grown with no competitive treatment (10 individuals * 3 soils * 4 reps), to a grand total of 324 pots. The first experimental Phase (Phase 1 hereafter) demonstrated plant growth in the field‐collected soils. After the harvest of Phase 1, the experiment was conducted again (Phase 2), sowing into the same pots, where the same competitive arrangement was planted into the corresponding pot, such that the pot was conditioned by the plants grown in Phase 1. No additional soil or sterilization was applied prior to Phase 2, as such the soils in Phase 2 reflect the field soil and the conditioning effects of Phase 1. Since nutrient availability and soil biota were not controlled, Phase 2 results are indicative of soil legacy, and do not isolate the mechanisms of PSF like soil microbial interactions. Phase 1 was conducted in January–March 2022, and Phase 2 in April–June 2022. The chronological design demonstrated how prior plant growth influences subsequent plant performance (Phase 2). Pots were organized into groups corresponding to the replicate and soil combinations (replicate groups) and placed throughout the greenhouse benches without fixed spatial clusters. These groups were shifted weekly across bench space so that each group experienced similar environmental conditions over the length of the experiment.

### 2.2 Statistical Methods

All statistical analyses were performed in R (R Core Team 2024). To determine the effects of density on per capita biomass, we approached the analysis with two model structures. First, we used a series of generalized linear mixed effects models which allowed us to capture direct slopes and interactions among fixed effects. Then we used non‐linear competition models based on the work of Spitters (1983) and Firbank and Watkinson (1985) to discern intraspecific and interspecific competition coefficients using our addition series design. These different model structures addressed our questions by using different aspects of the data, and their parameterizations are not directly comparable; thus, we will report the results separately.

#### 2.2.1 Linear Mixed‐Effects Models

Analysis of Phase 1 was conducted separately from Phase 2. For Phase 1, linear mixed‐effects models of either *B. tectorum* or *V. dubia* biomass were fit with the predictors: soil origin (neutral, *B. tectorum* , or *V. dubia* ), *B. tectorum* density, *V. dubia* density, and their interactions, with the random effect of replicate group. Replicate group was used as the random effect to account for lack of independence because physical position of the replicate groups was rotated throughout the greenhouse space. For Phase 2, similar linear mixed‐effects models of either *B. tectorum* or *V. dubia* biomass were fit with the predictors: soil origin, *B. tectorum* density, *V. dubia* density, a new predictor Phase 1 Biomass, and their interactions, with the random effect of pot replicate. The predictor Phase 1 Biomass is the biomass of equivalent individual plants from the same pot from Phase 1 of the experiment (in Phase 2 the same competition arrangement was planted into the pots after removal of the previous aboveground biomass). This variable would clarify whether there were effects of soil conditioning from Phase 1 to Phase 2. Due to heteroscedasticity in the data for both Phases, the biomass response variable was transformed using the natural logarithm. Residual diagnostics indicated our data were disproportionately influenced by observations with extreme residuals. We therefore used a consistent 1.5 times the interquartile range (IQR) criterion (Tukey's fence) to identify observations with extreme model residuals (Supplemental Table S1a). We conducted the analysis with and without these outliers to assess the sensitivity of the results to outlier removal. The patterns (direction and magnitude) were similar between analyses; therefore, we retained the models without outliers due to better residuals and these results are shown here. All two‐way and three‐way interactions were included in the full model and assessed using analysis of variance (ANOVA). Non‐significant interactions were removed through stepwise model selection to obtain the most parsimonious final models. All additive fixed effects were retained regardless of significance due to their roles in testing a priori hypotheses. A post hoc Tukey–Kramer test (*α* = 0.05) was performed using the emmeans package (Russell 2024) to determine pairwise differences between soil origins.

#### 2.2.2 Non‐Linear Competition Models

To determine competitive effects for both Phase 1 and Phase 2 (separately), analysis of each species' biomass was performed after methods by Spitters (1983) and Firbank and Watkinson (1985). Monoculture density data were used to determine intraspecific competition, and density and proportion in a biculture were used for interspecific competition. Analyses were performed using non‐linear regression to provide competition coefficients for each soil origin. Models were fit with a natural logarithmic transformation (Cousens 1991). As with our linear analysis, residual diagnostics indicated our data were disproportionately influenced by observations with extreme residuals; thus, we removed observations with residuals that were outside of 1.5 times the IQR (Supplemental Table S1b) as above. Again, we repeated sensitivity tests to assess the results to outlier removal. The patterns (direction and magnitude) were similar between analyses; therefore, we again retained models without outliers and these results are shown here.

We first determined the mean per capita biomass when grown at increasing density in monoculture. Mean biomass per plant (w) can be described by the Equation (1) using the natural logarithm:

(1) $$\ln\left(w\right)=\ln\left(w_{m}\right)-b*\ln\left(1+\mathrm{\alpha N}\right)$$

where $w_{m}$ was the mean biomass of individuals grown alone, a was the area encircling the individual required to reach the biomass $w_{m}$, N was the total density of plants at harvest, and b was the mean species‐specific resource use efficiency (Firbank and Watkinson 1985; Cousens 1991). We then determined mean biomass per plant when grown at increasing density of conspecifics and allospecifics, where mean biomass per plant of *B. tectorum* ($w_{B})$ or *V. dubia* ($w_{V}$) can be described by the equations:

(2) $$\ln\left(w_{B}\right)=\ln\left(w_{\mathrm{mB}}\right)-b_{B}*\ln\left(1+\beta_{B}\left(N_{B}+\alpha_{\mathrm{BV}}N_{V}\right)\right)$$

(3) $$\ln\left(w_{V}\right)=\ln\left(w_{\mathrm{mV}}\right)-b_{V}*\ln\left(1+\beta_{V}\;\left(N_{V}+\alpha_{\mathrm{VB}}N_{B}\right)\right)$$

where $w_{\mathrm{mB}}$ was the mean biomass of *B. tectorum* individuals grown alone, $\beta_{B}$ was the slope of *B. tectorum* intraspecific competition, $\beta_{V}$ was the slope of *V. dubia* intraspecific competition, $\alpha_{\mathrm{BV}}$ was the competition coefficient of the impact of *V. dubia* on *B. tectorum* , $\alpha_{\mathrm{VB}}$ was the competition coefficient of the impact of *B. tectorum* on *V. dubia* , $N_{B}$ was the density of *B. tectorum* at harvest, $N_{V}$ was the density of *V. dubia* at harvest, and $b_{B}$ was thought to be the mean species‐specific resource use efficiency of *B. tectorum* and $b_{V}$ for *V. dubia* .

The parameters α and b from Equation (1) were used in Equation (2) and Equation (3), where we were able to determine the intraspecific and interspecific competition coefficients for each species (β and α, respectively). To determine if there were differences in competition coefficients, bootstrapping was used to estimate confidence intervals. We resampled the dataset with replacement 1000 times before then refitting the model to each resampled dataset and extracting the competition coefficients for comparison.

## 3 Results

### 3.1 Phase 1

#### 3.1.1 Effects of Field Soil Origin and Density on Per Capita Biomass

Density affected both species while soil origin affected *B. tectorum* more than *V. dubia*. We observed a difference in *B. tectorum* per capita biomass in relation to soil origin (*p* < 0.001) as well as interactions between soil origin and *B. tectorum* density (*p* = 0.022) and *V. dubia* density (*p* = 0.062; Tables S2a and S3a). *Bromus tectorum* per capita biomass was highest in the *V. dubia* soil (0.112 g), followed by the *B. tectorum* soil, though there was very weak evidence they differed (0.094 g, *p* = 0.080), and by the neutral soil (0.063 g) which differed significantly from the *V. dubia* soil (*p* < 0.001), but only marginally differed from *B. tectorum* (*p* = 0.064; Tables 1a and S6a). *Ventenata dubia* per capita biomass did not show evidence of an interaction between or among soil origin or density of *B. tectorum* or *V. dubia* (*p* > 0.127, all combinations; Tables S4a and S5a). *Ventenata dubia* per capita biomass was greatest in the *V. dubia* soil (0.020 g) and *B. tectorum* soil (0.018 g) with no statistical difference between the two (*p* = 0.443). There was a decrease in biomass in the neutral soil (0.014 g) relative to the *V. dubia* and *B. tectorum* soils (*p* < 0.001, *p* = 0.025; Tables 2a and S7a).

The per capita biomass of *B. tectorum* was impacted by the combined effect of soil origin and density of *B. tectorum* and *V. dubia* (*p* = 0.022, *p* = 0.062 respectively; Table S3a). Intraspecific competition decreased per capita biomass of *B. tectorum* when grown in the neutral soil, such that each additional *B. tectorum* plant led to a 15% decrease in biomass (*p* < 0.001). The effect was weaker in the *B. tectorum* soil, as the decrease in biomass was 11% per additional *B. tectorum* plant (*p* = 0.006) and non‐significant in the *V. dubia* soil (*p* = 0.357; Table 1a, Figure 2a). Every added *V. dubia* led to a 9% decrease in per capita biomass of *B. tectorum* when grown in *V. dubia* soil (*p* = 0.030). This effect was weaker in the neutral soil, where biomass decreased by 3% for each added *V. dubia* individual (*p* = 0.021), and non‐significant in the *B. tectorum* soil (*p* = 0.909; Table 1a; Figure 2b).

**TABLE 1:** Model output of *B. tectorum* biomass given three soil origins and increasing density of *B. tectorum* and *V. dubia* during (a) Phase 1 and (b) Phase 2.

Fixed effects	Estimate	Standard error	Degrees of freedom	*t* value	*p*
**(a)**					
Intercept	−1.474	0.066	539.591	−22.31	< 0.001***
Soil *B. tectorum*	−0.126	0.101	453.256	−1.24	0.2151
Soil *V. dubia*	0.369	0.113	538.404	3.26	0.001***
*B. tectorum* count	−0.160	0.011	301.705	−14.55	< 0.001***
*V. dubia* count	−0.032	0.014	296.168	−2.33	0.0206**
Soil *B. tectorum* × *B. tectorum* count	0.045	0.016	212.703	2.76	0.0063**
Soil *B. tectorum* × *V. dubia* count	0.002	0.019	268.149	0.11	0.9091
Soil *V. dubia* × *B. tectorum* count	0.016	0.017	457.267	0.92	0.3574
Soil *V. dubia* × *V. dubia* count	−0.061	0.028	266.996	−2.18	0.0303*
**(b)**					
Intercept	−1.793	0.1563	242.3	−11.468	< 0.001***
Soil *B. tectorum*	−0.746	0.2005	219.2	−3.722	< 0.001***
Soil *V. dubia*	−0.247	0.2916	120.9	−0.847	0.399
*B. tectorum* count	−0.246	0.0204	512.8	−12.070	< 0.001***
*V. dubia* count	−0.150	0.0411	150.7	−3.633	< 0.001***
Phase 1 biomass	0.130	0.1295	5654.0	0.812	0.417
Soil *B. tectorum* × *B. tectorum* count	0.241	0.0300	260.9	8.037	< 0.001***
Soil *B. tectorum* × *V. dubia* count	0.180	0.0757	85.2	2.382	0.019**
Soil *V. dubia* × *B. tectorum* count	0.095	0.0462	911.0	2.059	0.042*
Soil *V. dubia* × *V. dubia* count	0.005	0.0080	117.6	0.061	0.952
Soil *B. tectorum* × *B. tectorum* count × *V. dubia* count	−0.038	0.0150	67.5	−2.518	0.014**
Soil *V. dubia* × *B. tectorum* count × *V. dubia* count	−0.015	0.0124	78.9	−1.214	0.228

*Note:* Asterisk next to *p*‐value indicates significance level (*p ≤ 0.050, **p < 0.025, ***p < 0.002).

**FIGURE 2:**
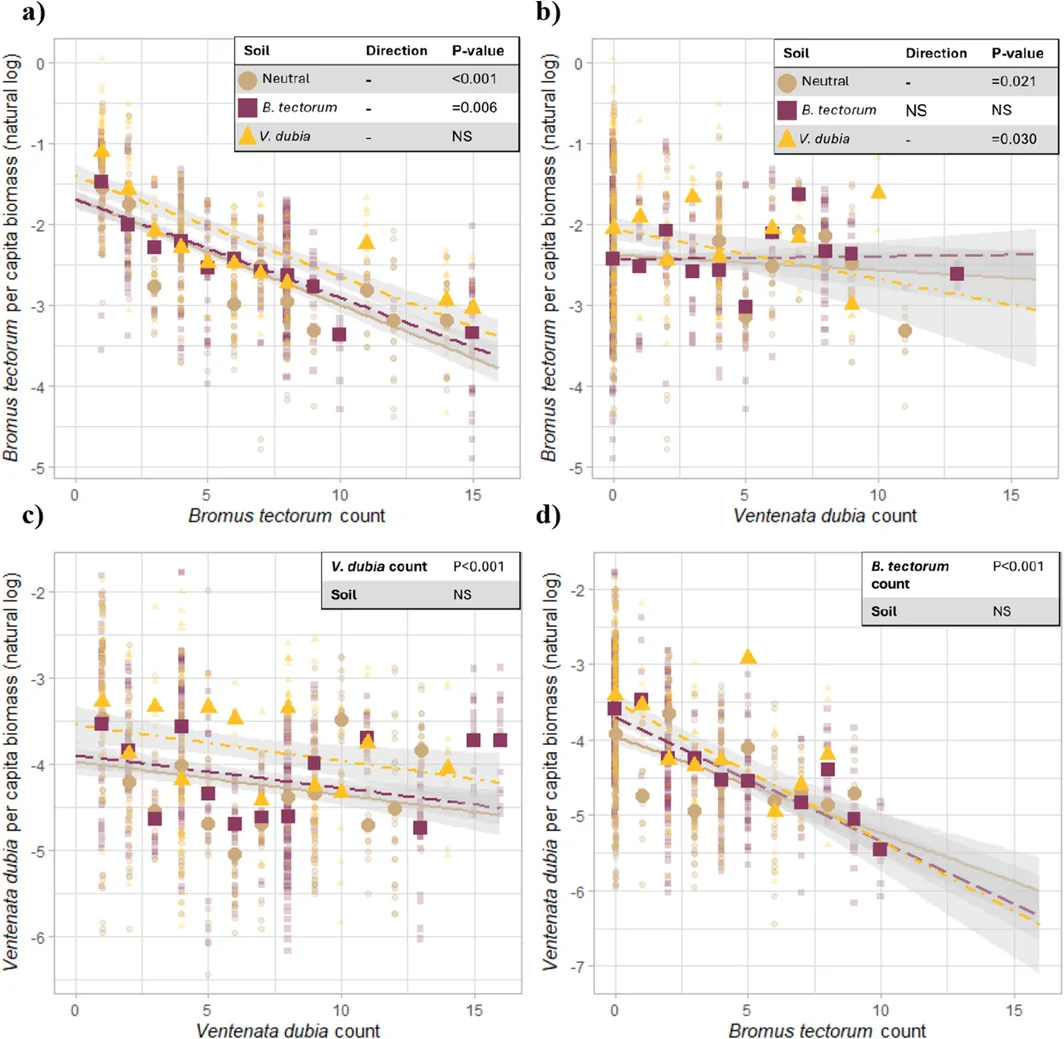
Phase 1 per capita biomass (natural logarithmically transformed) of (a) *Bromus tectorum* given count of *B. tectorum* , (b) *B. tectorum* given count of *V. dubia* , (c) *Ventenata dubia* given count of *V. dubia* and (d) *V. dubia* given count of *B. tectorum* . Lines represent three soil treatments (Neutral, *B. tectorum* soil, and *V. dubia* soil), where 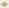 represents Neutral soil, 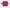
represents *B. tectorum* soil, and represents *V. dubia* soil. The upper right corner of each panel has a table indicating whether there was a significant interaction between soil type and density, and the direction of the interaction, where—represents a decrease, and = no change. If no interaction between density a particular soil and was detected, this was indicated by “NS” for “non‐significant.” In the instance that there was no interaction between soil and density, the different soil types were not listed, but the *p*‐value associated with the density effect was listed.

*Ventenata dubia* per capita biomass decreased as *B. tectorum* density and *V. dubia* density increased, irrespective of soil origin (Tables S4a and S5a). With intraspecific competition, every added *V. dubia* individual decreased mean per capita biomass by 4% (*p* < 0.001); whereas every additional *B. tectorum* individual decreased mean *V. dubia* per capita biomass by 15% on average (*p* < 0.001; Table 2a, Figure 2c,d).

**TABLE 2:** Model output of *V. dubia* biomass given three soil origins and increasing density of *Bromus tectorum* and *V. dubia* during (a) Phase 1 and (b) Phase 2.

Fixed effects	Estimate	Standard error	Degrees of freedom	*t* value	*p*
**(a)**					
Intercept	−3.627	0.07334	313.87016	−49.461	< 0.001***
Soil *B. tectorum*	0.22167	0.08697	231.70775	2.549	0.011**
Soil *V. dubia*	0.34695	0.10235	261.29191	3.390	< 0.001***
*B. tectorum* count	−0.16789	0.01597	199.57456	−10.511	< 0.001***
*V. dubia* count	−0.03951	0.01023	170.60247	−3.861	< 0.001***
**(b)**					
Intercept	−3.764	0.236	190.6	−15.940	< 0.001***
Soil *B. tectorum*	−0.507	0.370	127.9	−1.370	0.173
Soil *V. dubia*	−0.003	0.443	110.0	−0.007	0.994
*B. tectorum* count	−0.550	0.032	578.6	−17.225	< 0.001***
*V. dubia* count	−0.023	0.048	418.90	−0.486	0.627
Phase 1 biomass	0.032	0.160	4527.0	0.199	0.842
Soil *B. tectorum* × *B. tectorum* count	0.352	0.068	101.00	5.152	< 0.001***
Soil *B. tectorum* × *V. dubia* count	−0.154	0.067	174.9	−2.285	0.024**
Soil *V. dubia* × *B. tectorum* count	0.172	0.064	134.7	2.667	0.008**
Soil *V. dubia* × *V. dubia* count	0.008	0.064	171.6	0.126	0.899
*B. tectorum* count × *V. dubia* count	0.034	0.009	1410.0	3.842	0.001***

*Note:* Asterisk next to *p*‐value indicates significance level (*p ≤ 0.050, **p < 0.025, ***p < 0.002).

### 3.2 Phase 2

#### 3.2.1 Effects of Conditioned Soil Origin and Density on Per Capita Biomass

During Phase 2, both species were affected by interactions between conditioned soil origin and density. We observed interactions between soil origin and *B. tectorum* density (*p* < 0.001), *B. tectorum* density and *V. dubia* density (*p* = 0.058), and a three‐way interaction between soil origin, *B. tectorum* density, and *V. dubia* density (*p* = 0.038; Table S2b). However, we observed no difference in *B. tectorum* per capita biomass due to soil origins alone (*p* = 0.156), Phase 1 biomass (*p* = 0.417), nor interaction between soil origin and *V. dubia* density or any interactions with Phase 1 biomass (*p* > 0.16, all; Tables S2b, S3b and S5b). However, we observed *V. dubia* per capita biomass was impacted by soil origin (*p* = 0.005), as well as interactions between soil origin and *B. tectorum* density (*p* < 0.001) and *V. dubia* density (*p* = 0.031), and as with *B. tectorum* , Phase 1 biomass did not have an impact (*p* = 0.843; Tables S3b and S4b). *Ventenata dubia* per capita biomass was greatest in the *V. dubia* soil (0.014 g) but did not differ from the neutral soil (0.008 g, *p* = 0.247), and was least in the *B. tectorum* soil (0.004 g, *p* = 0.002), though there was no difference between the neutral and *B. tectorum* soil (*p* = 0.179, Tables 2b and S7b).

The per capita biomass of *B. tectorum* was impacted by the combined effect of soil origin and density of *B. tectorum* (*p* < 0.001) and *V. dubia* (*p* < 0.001), but not the combined effect of soil origin and *V. dubia* density (*p* = 0.196; Tables S2b and S3b). Intraspecific competition was most apparent in the neutral soil where each additional *B. tectorum* plant decreased biomass by 22% (*p* < 0.001); this effect was weaker in *V. dubia* soil (14% decrease; *p* = 0.016). Notably, in *B. tectorum* conditioned soil, there was < 1% change in biomass with increasing *B. tectorum* density (*p* < 0.001; Table 1b, Figure 3a,b) suggesting little density dependence. Interspecific competition showed *V. dubia* density affected *B. tectorum* per capita biomass when controlling for the effect of soil origin; each additional *V. dubia* plant led to a 14% decrease in *B. tectorum* biomass (*p* < 0.001), however this negative effect was weakened at higher *B. tectorum* densities, as indicated by the positive interaction between *B. tectorum* and *V. dubia* density (*p* = 0.058; Tables 1b and S3b). The strength of the three‐way density and soil interaction (*p* = 0.038, Table S3b) was most apparent in the *B. tectorum* soil where the negative relationship between *V. dubia* density and *B. tectorum* biomass became stronger again at higher *B. tectorum* densities. The combined effect of increasing density for both *B. tectorum* and *V. dubia* in the *B. tectorum* soil led to an additional 3.7% decrease in *B. tectorum* biomass per added *V. dubia* beyond what would be expected from the two‐way interaction alone (*p* = 0.014; Table 1b, Figure 3b).

**FIGURE 3:**
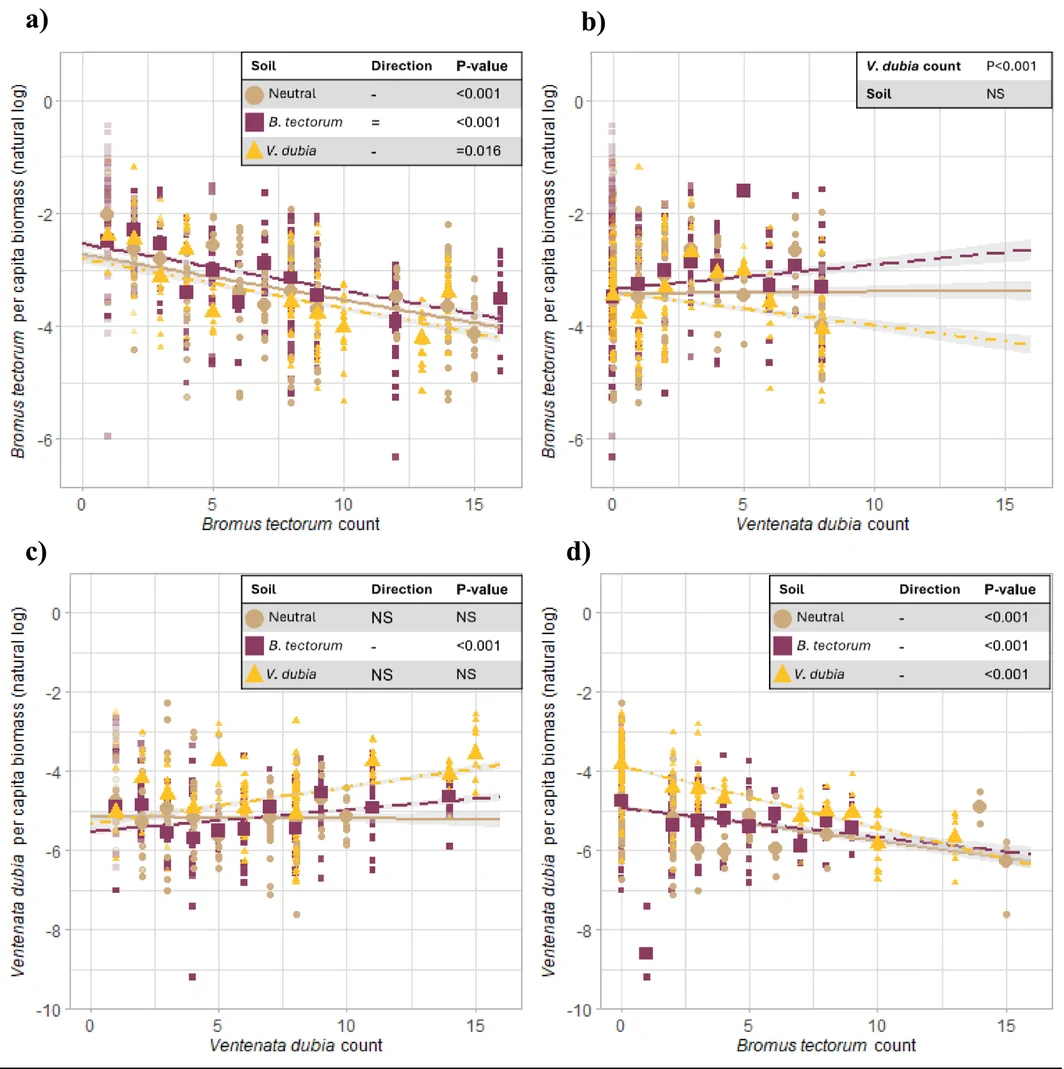
Phase 2 per capita biomass (natural logarithmically transformed) of (a) *Bromus tectorum* given count of *B. tectorum* , (b) *B. tectorum* given count of *V. dubia* , (c) *Ventenata dubia* given count of *V. dubia* and (d) *V. dubia* given count of *B. tectorum* . Lines represent three soil treatments (Neutral, *B. tectorum* soil, and *V. dubia* soil), where 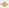 represents Neutral soil, 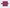
represents *B. tectorum* soil, and 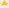 represents *V. dubia* soil. The upper right corner of each panel has a table indicating whether there was a significant interaction between soil type and density. If no interaction between density a particular soil and was detected, this was indicated by “NS” for “non‐significant.” In the instance that there was no interaction between soil and density, the different soil types were not listed, but the *p*‐value associated with the density effect was listed.

*Ventenata dubia* per capita biomass was impacted by soil origin interactions with the densities of *B. tectorum* and *V. dubia* (*p* < 0.001, *p* = 0.031; Tables S4b and S5b). Intraspecific competition was not significant in the neutral and *V. dubia* soils (*p* = 0.627, *p* = 0.899; Table 2b). However, in the *B. tectorum* soil every additional *V. dubia* plant led to a decrease in *V. dubia* biomass by 16% (*p* = 0.024; Table 2b, Figure 3c), suggesting that intraspecific competition became important when the soil was previously conditioned by *B. tectorum* , as opposed to soil conditioned by *V. dubia* . Interspecific competition had more effect: in neutral soil each additional *B. tectorum* plant was associated with a decrease in *V. dubia* biomass by 42% (*p* < 0.001). This effect was weaker—though still strong—in the *B. tectorum* and *V. dubia* soils, where for every additional *B. tectorum* plant *V. dubia* biomass decreased by 18% and 32% respectively (*p* < 0.008, both; Table 2b, Figure 3d). When controlling for the effect of soil, *V. dubia* biomass decreased with increasing *B. tectorum* density (*p* < 0.001), but the strength of this effect was weakened at higher *V. dubia* densities, as indicated by the positive interaction between *B. tectorum* and *V. dubia* density (*p* < 0.001, Table 2b).

#### 3.2.2 Intraspecific and Interspecific Competition on Per Capita Biomass

Competition coefficients from the non‐linear models showed few differences in competition strength across soil origins and experimental Phases. For both *B. tectorum* and *V. dubia* intraspecific competition ($\beta_{B}$ and $\beta_{V}$) was generally greater than interspecific competition, however the values did not significantly differ within species between soils (confidence intervals overlapped). However, *B. tectorum* 's coefficients were consistently smaller than *V. dubia* (Tables 3 and S8) suggesting it was less impacted by competition than *V. dubia* .

**TABLE 3:** Bootstrap estimates of non‐linear analysis of the natural log transformed biomass of either *B. tectorum* or *V. dubia* given intraspecific (β) and interspecific (α) competition. Dataset was resampled 1000 with replacement.

Phase	Species	Soil	β	95% Confidence interval	α	95% Confidence interval
1	*V. dubia*	Neutral	1.604	1.252–2.017	1.495	0.835–2.382
		*V. dubia*	1.740	1.349–2.195	1.546	0.858–2.463
		*B. tectorum*	1.707	1.325–2.152	1.534	0.852–2.444
	*B. tectorum*	Neutral	0.703	0.627–0.780	0.27	0.131–0.449
		*V. dubia*	0.526	0.471–0.582	0.295	0.157–0.469
		*B. tectorum*	0.609	0.545–0.675	0.282	0.144–0.458
2	*V. dubia*	Neutral	1.547	1.210–1.942	1.474	0.826–2.342
		*V. dubia*	1.839	1.422–2.326	1.582	0.879–2.530
		*B. tectorum*	1.618	1.262–2.035	1.501	0.837–2.391
	*B. tectorum*	Neutral	0.695	0.620–0.771	0.271	0.132–0.450
		*V. dubia*	0.711	0.634–0.789	0.269	0.130–0.449
		*B. tectorum*	0.783	0.697–0.870	0.261	0.122–0.443

For *B. tectorum* in Phase 1, the impact of *V. dubia* on *B. tectorum* ($\alpha_{\mathrm{BV}}$) was consistently lower regardless of soil, relative to the impact of *B. tectorum* on *V. dubia* ($\alpha_{\mathrm{VB}}$). The highest value observed of *V. dubia* impact on *B. tectorum* was in *V. dubia* soil during Phase 1 ($\alpha_{\mathrm{BV}}$ = 0.295), while the greatest impact of *B. tectorum* on *V. dubia* was in *V. dubia* conditioned soil in Phase 2 ($\alpha_{\mathrm{VB}}$ = 1.582; Tables 3 and S8). *Ventenata dubia* 's competitive strength against *B. tectorum* decreased in *V. dubia* soil between Phases 1 and 2, as *B. tectorum* biomass was more impacted in Phase 1 *V. dubia* soil ($\alpha_{\mathrm{BV}}=0.295$) relative to Phase 2 *V. dubia* soil ($\alpha_{\mathrm{BV}}=0.269$). This pattern was the same in the *B. tectorum* soil; however, in the neutral soil *B. tectorum* biomass was more impacted in Phase 2 ($\alpha_{\mathrm{BV}}=0.271$) relative to Phase 1 ($\alpha_{\mathrm{BV}}=0.270$; Tables 3 and S8).

## 4 Discussion

### 4.1 Competitive Interactions

We observed that intraspecific competition negatively impacted both species in both Phases, but by Phase 2 competition was weakened in conspecific soil, suggestive of a positive PSF. In addition, competition coefficients from the non‐linear analysis did not vary within species among soil types, but indicated that intraspecific competition was always greater than interspecific. In Phase 1, both species had the same biomass response in *B. tectorum* and *V. dubia* soils (both larger in these soils relative to neutral, with no difference between the two) but diverged in Phase 2. In Phase 2, *V. dubia* biomass was significantly larger in the *V. dubia* soil and smallest in the *B. tectorum* soil: this may be indicative of a negative effect of *B. tectorum* conditioned soil on *V. dubia* , not supporting our hypothesis. However, in Phase 2, *B. tectorum* had no differences among soil origins, suggesting that effects of soil legacy are weak or context dependent, again not supporting our hypotheses.

In a previous addition series study with *B. tectorum* and *V. dubia* , both species had greater aboveground biomass responses to interspecific competition than intraspecific competition (Sencenbaugh et al. 2025). The finding of greater impact of interspecific competition in this previous study contradicts our current bootstrapped mean competition coefficients, which showed that intraspecific competition had a greater influence on mean per capita aboveground biomass than interspecific competition for both species. Evaluating this further, we found that when grown in conspecific soils, intraspecific competition had less impact from Phase 1 to Phase 2, such that per capita biomass stabilized (i.e., showed some density dependence). Intraspecific competition coefficients were not statistically different from one another within species and soil origin, though intraspecific competition was consistently greater than interspecific. These results may seem contradictory to our linear model findings in Phase 2 where *B. tectorum* intraspecific competition caused < 1% difference in biomass in *B. tectorum* soil when there was a decrease in biomass in other soils. This apparent misalignment stems from the hypothesis tests addressed by the linear mixed effects models and the non‐linear competition models. The linear mixed effects models test for slope differences in the per capita biomass response given the fixed effects, whereas the non‐linear competition model estimates competition parameters were based on the addition series framework (Spitters 1983; Firbank and Watkinson 1985) where the *b* parameter is assumed to be −1, thus both species are assumed to have the same resource use efficiency. These two approaches therefore highlight different assumptions in the analysis of the data, but they agree that increasing density negatively influences biomass. While competition coefficients did not differ based on soil, the explanatory power of soil origin in our linear mixed effects models was significant, and the influence of PSF should not be altogether dismissed.

### 4.2 PSF And Competition

Considering plant–soil interactions in the context of competition, intraspecific competition impacted both species: in Phase 1, *B. tectorum* was less impacted in its own soil while *V. dubia* did not differ based on soil origin, whereas in the conditioned soils of Phase 2, intraspecific competition was weaker for both species, especially in their own soils. This pattern suggests positive PSF, where the plants may have modified their own soil microbial community or environment in a way that promotes their own species growth, allowing for more conspecific individuals to live in higher densities (Bever 1994; Bever et al. 2015). At larger spatial scales, invasive species have demonstrated negative feedbacks in their native range, but not in the invaded range (Reinhart et al. 2003; Dostálek et al. 2016). The lack of negative feedback in the invaded range may be due to escape from soil microbes or pathogens, which could lead to competitive advantages over native species in the invaded communities (Reinhart et al. 2003; Dostálek et al. 2016).

PSF may change competitive interactions between co‐occurring invasive species and therefore their invasion dynamics (Chisholm and Muller‐Landau 2011; Crawford and Knight 2017; Zhang et al. 2020). The scenario in which one invader promotes the fitness of other invaders can be detrimental to management (Simberloff and Von Holle 1999). Contrastingly, competitive interactions between invaders can limit their populations and decrease their impact on the entire plant community (Rauschert and Shea 2012, 2017; Brandt et al. 2023). Chen and van Kleunen (2022) found positive soil legacy effects between invaders, where invaders benefited from soil where a previous invader of a different species had grown, likely due to plant‐caused changes in the soil microbial community. Climate change models of the American West predict increased habitat suitability for *B. tectorum* into the northern and eastern sections of the sagebrush biome (Bradley 2009; Taylor et al. 2014). Similarly, suitable habitat for *V. dubia* is expected to increase due to heating and drying summer conditions (Adhikari et al. 2022; Chang et al. 2023; Nietupski et al. 2024). These range shifts will amplify the range overlap between these two species, increasing the opportunity for interactions. While *B. tectorum* demonstrated greater competitive effects on *V. dubia* in our work, *V. dubia* has demonstrated neutral or stronger competitive effects relative to *B. tectorum* in other studies (Harvey et al. 2020; Sencenbaugh et al. 2025).

Positive PSF are generally not conducive to coexistence of multiple species due to the loss of density dependence and greater biculture interspecific limitation (Chesson 2000; Barabás et al. 2018; Zhang et al. 2020). The mechanisms through which negative PSF are thought to promote coexistence are through increased niche partitioning and greater intraspecific competition relative to interspecific competition (Chesson 2000; Barabás et al. 2018; Ke and Wan 2020). A meta‐analysis by Crawford et al. (2019) found that most PSF relationships are negative, with greater negative impacts in field studies than in greenhouse studies (though they found far fewer field studies relative to greenhouse). Negative PSF indicate many plants demonstrate density dependence, thus promoting greater diversity and coexistence (Chesson 2003; Chisholm and Muller‐Landau 2011; Bever et al. 2015; Barabás et al. 2018; Crawford et al. 2019). Positive PSF, along with other factors like dispersal and habitat availability, may explain clumped distributions of plants or dense monoculture stands (Bennett et al. 2017), which are often observed in invasive species (Crawford and Knight 2017).

*Bromus tectorum* was least impacted by interspecific competition in its own soil and most impacted in *V. dubia* soil in Phase 1. This difference in response to competition in different soil origins may be indicative of positive *B. tectorum* PSF, and/or negative effects of *V. dubia* conditioned soil on *B. tectorum*. Both *B. tectorum* and *V. dubia* are known to grow in dense monocultures in areas that are heavily disturbed (Gasch et al. 2013; Germino et al. 2015; Fryer 2017; Jones et al. 2018), and in patches where there is less disturbance or where they are at the edge of their realized niche (Jones et al. 2018; Peeler and Smithwick 2018). It is possible that positive PSF acting within a monoculture patch may provide an advantage to a species that could lead to the exclusion of the other species (Crawford and Knight 2017) and help populations expand (Bever et al. 1997; Suding et al. 2013).

### 4.3 Potential Secondary Invasion

Though *B. tectorum* and *V. dubia* do co‐occur, the association can be positive or negative due to their differing abiotic/niche requirements, where often *V. dubia* dominates cooler and wetter microsites, while *B. tectorum* dominates drier sites (Jones et al. 2018, 2020; Applestein and Germino 2022). However, associations between *V. dubia* and other invasive and native grasses indicate *V. dubia* has a wide range of soil moisture tolerances (Jones et al. 2020) which could allow for greater overlap with *B. tectorum* as climate continues to change. In general, we found *B. tectorum* density had a greater impact on *V. dubia* biomass than *V. dubia* density had on *B. tectorum* biomass. This contradicts our hypothesis that neither species would have an obvious advantage over the other based on previous work with these species (Harvey et al. 2020; Sencenbaugh et al. 2025). In this study, pots were watered every other day to maintain somewhat dry, but sufficient moisture for germination and survival for both species. Competitive interactions observed reflect this watering regime, where under different watering conditions interactions may shift. Future research should address these interactions under varying abiotic conditions (i.e., temperature and watering) to better understand how these relationships may shift under future climate change.

Our finding that *B. tectorum* had a greater impact on *V. dubia* than vice versa would suggest an interaction between these species where *B. tectorum* demonstrates competitive dominance over *V. dubia* under our study conditions. However, this relationship became less clear by Phase 2 of our experiment, where there was a mediating effect of conspecific density. For both species, the impact of allospecific individuals decreased as the density of conspecifics increased. This may suggest that in a community with a high density of *V. dubia* , *B. tectorum* invasion would have less impact on a single *V. dubia* plant than it would in a community with fewer established *V. dubia* individuals. As such, *B. tectorum* is not likely to invade a community dominated by *V. dubia* unless management or disturbance alters the existing *V. dubia* population.

The impacts of interspecific competition should be considered through the lens of secondary invasion, as the documented competitive effects of these species on one another could be conducive to secondary invasion (Pearson, Ortega, Runyon, and Butler 2016). If a dominant invader was removed from the community, the subdominant invader may be released from competitive pressure, allowing for a consequent increase in population density or range (Pearson, Ortega, Runyon, and Butler 2016; Oduor et al. 2024). Often secondary invaders differ from primary invaders in growth form or life‐history strategy, where management impacts the dominant invader but not the subdominant (Pearson, Ortega, Eren, and Hierro 2016; Pearson, Ortega, Runyon, and Butler 2016), but these two species are similar. Both species germinate in the fall, and are thus managed using pre‐ or early post‐emergent herbicide application using active ingredients like imazapic and indaziflam (Mangold et al. 2013; Monaco et al. 2017; Sebastian et al. 2017). However, *V. dubia* has spring germinating cohorts in rangelands (Wallace et al. 2015). If management is done using herbicides without multi‐season residual activity (i.e., imazapic), this strategy may target *B. tectorum* and miss the spring *V. dubia* cohort, which may contribute to secondary invasion. The competitive effect of *B. tectorum* on *V. dubia* may suggest that in the field *B. tectorum* dominates *V. dubia* , but in the instance that management misses the spring cohort of *V. dubia* , perhaps secondary invasion could occur. Further, we observed that *V. dubia* biomass was greater in *V. dubia* conditioned soil (in Phase 1), which may be indicative of a positive PSF, conducive to the formation of a monoculture. While this was suggestive of *V. dubia* positive PSF, it is unlikely to overcome or outcompete *B. tectorum* where *B. tectorum* is abundant. Further, analysis of the soil microbial community and AM fungi associations with these species when co‐occurring is necessary before drawing any conclusions on the extent of potential PSF.

## 5 Conclusion

Under future climate change scenarios that favor *V. dubia* , *V. dubia* may demonstrate enhanced competitive effects. Harvey et al. (2020) found that under projected 2100 temperature and CO_2_, *V. dubia* and *B. tectorum* showed little evidence of aboveground competition, but potential belowground competition. Further, *V. dubia* demonstrated greater root to shoot ratios under elevated temperature and CO_2_, which may indicate greater water use efficiency, a response not demonstrated by *B. tectorum* (Harvey et al. 2020). Our study suggests these species may be self‐perpetuating over time through positive feedback with their own soils when grown in biculture. However, the mechanisms should be further investigated with consideration for field conditions, particularly varying abiotic conditions (temperature and moisture) and biotic interactions (especially with AM fungi). Further, the microbial organisms that may be contributing to any PSF of these species should be examined. Concurrently, one must be cautious about interpretations beyond the biculture context of these findings as well as the spatial and temporal scale at which microbial and physical processes interact with the plant community to produce different patterns of plant species composition. It is particularly important in co‐occurring invaders like *B. tectorum* and *V. dubia* to understand how species‐specific plant‐microbe interactions affect plant fitness and competitive ability, as these interactions may intensify negative impacts on native community composition.

## Author Contributions

**Lilly Sencenbaugh:** conceptualization (lead), data curation (lead), formal analysis (lead), methodology (equal), writing – original draft (lead), writing – review and editing (lead). **Jane M. Mangold:** conceptualization (equal), formal analysis (equal), methodology (equal), writing – review and editing (equal). **Bruce D. Maxwell:** conceptualization (supporting), formal analysis (supporting), methodology (supporting), writing – review and editing (equal). **Danielle E. M. Ulrich:** conceptualization (equal), formal analysis (equal), methodology (equal), writing – review and editing (equal). **Lisa J. Rew:** conceptualization (equal), formal analysis (equal), methodology (equal), writing – original draft (equal), writing – review and editing (equal).

## Funding

The authors have nothing to report.

## Conflicts of Interest

The authors declare no conflicts of interest.

## Supporting information


**Table S1:** Number of observations and outliers removed for (a) linear and (b) non‐linear models. Removed observations were associated with residuals that were outside of 1.5 times the interquartile range (IQR). Original observations are the number of observations at the end of the study, outliers removed are the number of extreme outliers removed, and final observations are the number of observations after outlier removal.
**Table S2:** Analysis of variance (ANOVA) output for full model of *B. tectorum* per capita biomass for (a) Phase 1 and (b) Phase 2. Boldened *p* values indicate statistical significance *p* ≤ 0.05.
**Table S3:** Analysis of variance (ANOVA) table for final selected model of the natural logarithmic transformed *B. tectorum* per capita biomass for (a) Phase 1 and (b) Phase 2.
**Table S4:** Analysis of variance (ANOVA) output for full model of *V. dubia* per capita biomass for (a) Phase 1 and (b) Phase 2.
**Table S5:** Analysis of variance (ANOVA) output for final model of the natural logarithmic transformed *V. dubia* per capita biomass for (a) Phase 1 and (b) Phase 2.
**Table S6:** Estimated marginal means of *B. tectorum* per capita biomass in (a) Phase 1 and (b) Phase 2 given soil type (Neutral, *B. tectorum* , or *V. dubia* ). Compact letter display (CLD) different if *p* ≤ 0.05.
**Table S7:** Estimated marginal means of *V. dubia* per capita biomass in (a) Phase 1 and (b) Phase 2 given soil type (Neutral, *B. tectorum* , or *V. dubia* ). Compact letter display (CLD) different if *p* ≤ 0.05.
**Table S8:** Model output of non‐linear analysis of the natural log transformed biomass of either *B. tectorum* or *V. dubia* given intraspecific (β) and interspecific (α) competition.

## Data Availability

The data that support the findings of this study are available from the online repository: https://datadryad.org/dataset/doi:10.5061/dryad.jwstqjqqz.
