# Supplementary material for: Plant–Soil Feedbacks May Underlie Competitive Dynamics Between Co‐Occurring Invasive Annual Grasses Bromus tectorum and Ventenata dubia
Source: Ecol Evol. 2026 Oct 18;16(10):e74516. doi: 10.1002/ece3.74516 (PMC13677447; doi:10.1002/ece3.74516)
Supplement: Supplementary file 1 [file ECE3-16-e74516-s001.docx]

Supplementary information

**Equations**

The natural log transformed mean yield per plant ($w$) can be described by the equation:

$$\ln\left( w \right)=\ln\left( w_{m} \right)-b*\ln\left( 1+\alpha N \right)$$

(1)

where $w_{m}$ was the mean biomass of individuals grown alone, $a$ was the area required to reach the biomass $w_{m}$, $N$ was the total density of plants at harvest, and $b$ was thought to be the mean species-specific resource use efficiency (Watkinson, 1985).

The natural log transformed mean biomass per plant of *B. tectorum* ($w_{B})$or *V. dubia* ($w_{V}$) can be described by the equations:

$$\ln\left( w_{B} \right)=\ln\left( w_{mB} \right)-b_{B}*\ln\left( 1+\beta_{B}\left( N_{B}+\alpha_{BV}N_{V} \right) \right)$$

(2)

$$\ln\left( w_{V} \right)=\ln\left( w_{mV} \right)-b_{V}*\ln\left( 1+ \beta_{V} \left( N_{V}+\alpha_{VB}N_{B} \right) \right)$$

(3)

where $w_{mB}$ was the mean biomass of *B. tectorum* individuals grown alone, $\beta_{B}$ was the slope of *B. tectorum* intraspecific competition, $\beta_{V}$ was the slope of *V. dubia* intraspecific competition, $\alpha_{BV}$ was the competition coefficient of the impact of *V. dubia* on *B. tectorum,*  $\alpha_{VB}$ was the competition coefficient of the impact of *B. tectorum* on *V. dubia,* $N_{B}$ was the density of *B. tectorum* at harvest, $N_{V}$ was the density of *V. dubia* at harvest, and $b_{B}$ was thought to be the mean species-specific resource use efficiency of *B. tectorum* and $b_{V}$ for *V. dubia*. The parameters $\alpha$ and $b$ from Equation 1b were used in Equations 2b and 3b, where we were able to determine the intraspecific and interspecific competition coefficients for each species ($\beta$ and $\alpha$, respectively).

**Tables**

Supplemental Information Table **S1**: Number of observations and outliers removed for a) linear and b) non-linear models. Removed observations were associated with residuals that were outside of 1.5 times the interquartile range (IQR). Original observations are the number of observations at the end of the study, outliers removed are the number of extreme outliers removed, and final observations are the number of observations after outlier removal.

**a)**

| **Phase** | **Species** | **Soil** | **Original observations** | **Outliers removed** | **Final observations** |
| --- | --- | --- | --- | --- | --- |
| 1 | *Bromus tectorum* | Neutral | 419 | 17 | 402 |
|  |  | *B. tectorum* | 401 | 10 | 391 |
|  |  | *V. dubia* | 187 | 7 | 180 |
|  | *Ventenata dubia* | Neutral | 463 | 33 | 430 |
|  |  | *B. tectorum* | 462 | 10 | 452 |
|  |  | *V. dubia* | 192 | 4 | 188 |
| 2 | *B. tectorum* | Neutral | 199 | 9 | 191 |
|  |  | *B. tectorum* | 257 | 8 | 250 |
|  |  | *V. dubia* | 225 | 29 | 197 |
|  | *V. dubia* | Neutral | 155 | 19 | 137 |
|  |  | *B. tectorum* | 204 | 3 | 202 |
|  |  | *V. dubia* | 204 | 17 | 188 |

**b)**

| **Phase** | **Species** | **Model** | **Soil** | **Original observations** | **Outliers removed** | **Final observations** |
| --- | --- | --- | --- | --- | --- | --- |
| 1 | *Bromus tectorum* | Monoculture | Neutral | 225 | 31 | 194 |
|  |  |  | *B. tectorum* | 204 | 11 | 193 |
|  |  |  | *V. dubia* | 106 | 14 | 92 |
|  |  | Biculture | Neutral | 419 | 21 | 398 |
|  |  |  | *B. tectorum* | 401 | 16 | 385 |
|  |  |  | *V. dubia* | 187 | 18 | 169 |
|  | *Ventenata dubia* | Monoculture | Neutral | 235 | 27 | 208 |
|  |  |  | *B. tectorum* | 202 | 13 | 189 |
|  |  |  | *V. dubia* | 112 | 6 | 106 |
|  |  | Biculture | Neutral | 463 | 56 | 407 |
|  |  |  | *B. tectorum* | 462 | 23 | 439 |
|  |  |  | *V. dubia* | 192 | 16 | 176 |
| 2 | *B. tectorum* | Monoculture | Neutral | 87 | 10 | 77 |
|  |  |  | *B. tectorum* | 140 | 8 | 132 |
|  |  |  | *V. dubia* | 106 | 17 | 89 |
|  |  | Biculture | Neutral | 199 | 9 | 190 |
|  |  |  | *B. tectorum* | 257 | 35 | 222 |
|  |  |  | *V. dubia* | 225 | 29 | 196 |
|  | *V. dubia* | Monoculture | Neutral | 77 | 0 | 77 |
|  |  |  | *B. tectorum* | 105 | 3 | 102 |
|  |  |  | *V. dubia* | 105 | 2 | 103 |
|  |  | Biculture | Neutral | 155 | 18 | 137 |
|  |  |  | *B. tectorum* | 204 | 19 | 185 |
|  |  |  | *V. dubia* | 204 | 17 | 187 |

Supplemental Information Table **S2**: Analysis of variance (ANOVA) output for full model of *B. tectorum* per capita biomass for **a)** Phase 1 and **b)** Phase 2. Boldened p values indicate statistical significance p < 0.05.

**a)**

| **Fixed effects** | **Chi-square** | **Degrees of freedom** | **p value** |
| --- | --- | --- | --- |
| Soil | 26.204 | 2 | **<0.001***** |
| B. tectorum count | 349.609 | 1 | **<0.001***** |
| V. dubia count | 18.411 | 1 | **<0.001***** |
| Soil × B. tectorum count | 7.699 | 2 | **0.021*** |
| Soil × V. dubia count | 5.541 | 2 | 0.063 |
| B. tectorum count × V. dubia count | 0.188 | 1 | 0.664 |
| Soil × B. tectorum count × V. dubia count | 1.214 | 2 | 0.545 |

**b)**

| **Fixed effects** | **Chi-square** | **Degrees of freedom** | **p value** |
| --- | --- | --- | --- |
| Soil | 5.887 | 2 | 0.053**`** |
| B. tectorum count | 126.564 | 1 | **<0.001***** |
| V. dubia count | 35.633 | 1 | **<0.001***** |
| Phase 1 biomass | 0.090 | 1 | 0.765 |
| Soil × B. tectorum count | 69.615 | 2 | **<0.001***** |
| Soil × V. dubia count | 2.377 | 2 | 0.305 |
| Soil × Phase 1 biomass | 1.885 | 2 | 0.390 |
| B. tectorum count × V. dubia count | 3.340 | 1 | 0.068` |
| B. tectorum count × Phase 1 biomass | <0.001 | 1 | 0.985 |
| V. dubia count × Phase 1 biomass | 0.497 | 1 | 0.481 |
| Soil × B. tectorum count × V. dubia count | 6.207 | 2 | **0.045*** |

**`** p = 0.051 - 0.070

***** p = 0.020 - 0.050

****** p = 0.001 - 0.019

******* p < 0.001

Supplemental Information Table S**3**: Analysis of variance (ANOVA) table for final selected model of the natural logarithmic transformed *B. tectorum* per capita biomass for **a)** Phase 1 and **b)** Phase 2.

**a)**

| Fixed effects | Chi-square | Degrees of freedom | p value |
| --- | --- | --- | --- |
| Soil | 26.2935 | 2 | <0.001*** |
| *B. tectorum* count | 353.4825 | 1 | <0.001*** |
| *V. dubia* count | 18.6360 | 1 | <0.001*** |
| Soil × *B. tectorum* count | 7.6250 | 2 | 0.022** |
| Soil × *V. dubia* count | 5.5563 | 2 | 0.062 |

**b)**

| Fixed effects | Chi-square | Degrees of freedom | p value |
| --- | --- | --- | --- |
| Soil | 3.7106 | 2 | 0.1564 |
| *B. tectorum* count | 109.3676 | 1 | <0.001*** |
| *V. dubia* count | 12.8219 | 1 | <0.001*** |
| Phase 1 biomass | 0.6594 | 1 | 0.4168 |
| Soil × *B. tectorum* count | 59.6683 | 2 | <0.001*** |
| Soil × *V. dubia* count | 3.2648 | 2 | 0.1955 |
| *B. tectorum* count × *V. dubia* count | 3.5921 | 1 | 0.0581* |
| Soil × *B. tectorum* count × *V. dubia* count | 6.5388 | 2 | 0.0380* |

Supplemental Information Table S**4**: Analysis of variance (ANOVA) output for full model of *V. dubia* per capita biomass for **a)** Phase 1 and **b)** Phase 2.

**a)**

| **Fixed effects** | **Chi-square** | **Degrees of freedom** | **p value** |
| --- | --- | --- | --- |
| Soil | 13.437 | 2 | **0.001**** |
| B. tectorum count | 105.491 | 1 | **<0.001***** |
| V. dubia count | 15.096 | 1 | **<0.001***** |
| Soil × B. tectorum count | 0.863 | 2 | 0.650 |
| Soil × V. dubia count | 2.036 | 2 | 0.361 |
| B. tectorum count × V. dubia count | 0.126 | 1 | 0.722 |
| Soil × B. tectorum count × V. dubia count | 3.134 | 2 | 0.209 |

**b)**

| **Fixed effects** | **Chi-square** | **Degrees of freedom** | **p value** |
| --- | --- | --- | --- |
| Soil | 10.748 | 2 | **0.005**** |
| B. tectorum count | 389.575 | 1 | **<0.001***** |
| V. dubia count | 0.212 | 1 | 0.645 |
| Phase 1 biomass | 0.039 | 1 | 0.843 |
| Soil × B. tectorum count | 29.630 | 2 | **<0.001***** |
| Soil × V. dubia count | 6.951 | 2 | **0.031*** |
| Soil × Phase 1 biomass | 0.021 | 2 | 0.989 |
| B. tectorum count × V. dubia count | 14.811 | 1 | **<0.001***** |
| B. tectorum count × Phase 1 biomass | 0.026 | 1 | 0.872 |
| V. dubia count × Phase 1 biomass | 0.072 | 1 | 0.788 |
| Soil × B. tectorum count× V. dubia count | 0.542 | 2 | 0.762 |

**`** p = 0.051 - 0.070

***** p = 0.020 - 0.050

****** p = 0.001 - 0.019

******* p < 0.001

Supplemental Information Table S**5**: Analysis of variance (ANOVA) output for final model of the natural logarithmic transformed *V. dubia* per capita biomass for **a)** Phase 1 and **b)** Phase 2.

**a)**

| Fixed effects | Chi-square | Degrees of freedom | p value |
| --- | --- | --- | --- |
| Soil | 13.406 | 2 | 0.001*** |
| *B. tectorum* count | 110.478 | 1 | <0.001*** |
| *V. dubia* count | 14.905 | 1 | <0.001*** |

**b)**

| Fixed effects | Chi-square | Degrees of freedom | p value |
| --- | --- | --- | --- |
| Soil | 10.6983 | 2 | 0.005*** |
| *B. tectorum* count | 390.6361 | 1 | <0.001*** |
| *V. dubia* count | 0.2138 | 1 | 0.645 |
| Phase 1 biomass | 0.0396 | 1 | 0.843 |
| Soil × *B. tectorum* count | 29.5375 | 2 | <0.001*** |
| Soil × *V. dubia* count | 7.0038 | 2 | 0.031** |
| *B. tectorum* count × *V. dubia* count | 14.7636 | 1 | <0.001*** |

Supplemental Information Table **S6**: Estimated marginal means of *B. tectorum* per capita biomass in **a)** Phase 1 and **b)** Phase 2 given soil type (Neutral, *B. tectorum,* or *V. dubia*). Compact letter display (CLD) different if p < 0.05.

**a)**

| **Soil** | **Estimated marginal mean** | **Standard error** | **Degrees of freedom** | **Compact letter display** |
| --- | --- | --- | --- | --- |
| Neutral | 0.0804 | 0.004 | 186 | a |
| Bromus tectorum | 0.0933 | 0.004 | 153 | ab |
| Ventenata dubia | 0.1116 | 0.008 | 205 | b |

**b)**

| **Soil** | **Estimated marginal mean** | **Standard error** | **Degrees of freedom** | **Compact letter display** |
| --- | --- | --- | --- | --- |
| Neutral | 0.022 | 0.003 | 134 | a |
| Bromus tectorum | 0.059 | 0.009 | 134 | a |
| Ventenata dubia | 0.029 | 0.005 | 97 | b |

Supplemental Information Table **S7**: Estimated marginal means of *V. dubia* per capita biomass in **a)** Phase 1 and **b)** Phase 2 given soil type (Neutral, *B. tectorum,* or *V. dubia*). Compact letter display (CLD) different if p < 0.05.

**a)**

| **Soil** | **Estimated marginal mean** | **Standard error** | **Degrees of freedom** | **Compact letter display** |
| --- | --- | --- | --- | --- |
| Neutral | 0.013 | 0.001 | 217 | a |
| Bromus tectorum | 0.017 | 0.001 | 206 | b |
| Ventenata dubia | 0.018 | 0.002 | 257 | b |

**b)**

| **Soil** | **Estimated marginal mean** | **Standard error** | **Degrees of freedom** | **Compact letter display** |
| --- | --- | --- | --- | --- |
| Neutral | 0.008 | 0.002 | 214 | ab |
| Bromus tectorum | 0.004 | 0.001 | 118 | a |
| Ventenata dubia | 0.014 | 0.003 | 113 | b |

Supplemental Information Table **S8**: Model output of non-linear analysis of the natural log transformed biomass of either *B. tectorum* or *V. dubia* given intraspecific ($\beta$) and interspecific ($\alpha)$ competition.

| **Phase** | **Target species** | **Soil** | $\boldsymbol{\beta}$ | **Standard error** | **p value** | $\boldsymbol{\alpha}$ | **Standard error** | **p value** |
| --- | --- | --- | --- | --- | --- | --- | --- | --- |
| 1 | *Bromus tectorum* | Neutral | 1.299 | 0.064 | <0.001 | 0.174 | 0.068 | =0.011 |
|  |  | *B. tectorum* | 0.592 | 0.030 | <0.001 | 0.280 | 0.076 | <0.001 |
|  |  | *V. dubia* | 1.155 | 0.067 | <0.001 | 0.500 | 0.130 | <0.001 |
|  | *Ventenata dubia* | Neutral | 7.898 | 0.526 | <0.001 | 1.326 | 0.239 | <0.001 |
|  |  | *B. tectorum* | 5.177 | 0.313 | <0.001 | 3.357 | 0.395 | <0.001 |
|  |  | *V. dubia* | 0.776 | 0.078 | <0.001 | 3.391 | 0.666 | <0.001 |
| 2 | *B. tectorum* | Neutral | 1.168 | 0.100 | <0.001 | 0.093 | 0.141 | =0.512 |
|  |  | *B. tectorum* | 3.087 | 0.249 | <0.001 | 0.070 | 0.111 | =0.528 |
|  |  | *V. dubia* | 1.888 | 0.136 | <0.001 | 0.220 | 0.124 | =0.079 |
|  | *V. dubia* | Neutral | 2.368 | 0.258 | <0.001 | 1.391 | 0.347 | <0.001 |
|  |  | *B. tectorum* | 1.214 | 0.153 | <0.001 | 2.091 | 0.563 | <0.001 |
|  |  | *V. dubia* | 0.368 | 0.048 | <0.001 | 7.041 | 1.332 | <0.001 |
